# Comment on “Lactylated histone H3K18 as a potential biomarker for the diagnosis and prediction of the severity of pancreatic cancer”

**DOI:** 10.1016/j.clinsp.2025.100729

**Published:** 2025-07-30

**Authors:** Rachana Mehta, Ranjana Sah

**Affiliations:** a SR Sanjeevani Hospital, Kalyanpur, Nepal; b Clinical Microbiology, RDC, Manav Rachna International Institute of Research and Studies, Faridabad, India; c Patil Vidyapeeth University Dr D Y Patil Medical College Hospital and Research Centre, Pune, India

Dear Editor,

We read with great interest the study by Hou et al., which proposes H3K18la as a putative biomarker for pancreatic cancer severity. The interrogation of epigenetic lactylation in oncopathology is a frontier area; however, this study’s design, interpretive framework, and translational extrapolations warrant critical scrutiny.

The investigators rely on a 21-patient single-center observational cohort, yet draw high-stakes diagnostic and prognostic implications. Such extrapolation lacks epidemiologic power. With only 12 subjects in the “High H3K18la” group and 9 in the “Low,” the statistical conclusions ‒ particularly subgroup analyses such as those correlating H3K18la expression with smoking (*p* = 0.0436), diabetes (*p* = 0.0436), and alcohol intake (*p* = 0.0195) ‒ are highly susceptible to type I errors. Notably, the authors perform no correction for multiple comparisons despite testing associations across 10 clinicopathologic variables. This omission inflates the apparent significance of several correlations and undermines the statistical reliability of the conclusions.

Moreover, the dichotomization of H3K18la into “High” and “Low” based on the cohort’s mean value (2.979 relative density) is methodologically imprecise. Biomarker thresholds should be empirically derived using external validation cohorts or optimized via Youden index from ROC analyses ‒ not retrospective mean-splitting within the test population^1^^,^^2^. This internal dichotomization risks overfitting and eliminates the capacity to detect dose-response gradients across the spectrum of H3K18la expression. Given that the authors report a strong correlation with CA19–9 (*r* = 0.744) and lactate (*r* = 0.774), multivariate regression including potential confounders such as tumor size, diabetes, and alcohol consumption would have been preferable to univariate correlation coefficients. Without such adjustments, the putative diagnostic value of H3K18la remains speculative.

From a biological standpoint, the interpretation of H3K18la as a driver of tumor progression rather than a passive epiphenomenon is unsubstantiated. H3K18 is a known transcriptional activation mark, but its lactylation (rather than acetylation) is a nascent area of study. While lactate-derived histone lactylation has been linked to immune polarization and macrophage activation, its role in cancer is context-dependent and bidirectional^3^^,^^4^. In renal carcinoma, lactate-fueled H3K18la has been shown to promote YTHDF2 transcription and downstream tumor growth, but such mechanistic pathways are not interrogated here^3^. The assertion that elevated H3K18la reflects “disease severity” lacks causal anchoring without gene expression profiling of downstream targets, ChIP-seq confirmation of locus specificity, or modulation of lactate dehydrogenase activity to test reversibility.

The translational ambitions are similarly overstated. While the AUC of 0.848 for H3K18la in serum is compelling, the absence of a control group precludes assessment of specificity. All 21 patients had pancreatic cancer, and no non-malignant controls (e.g., pancreatitis, biliary obstruction, or healthy donors) were included to distinguish cancer-specific expression. The biomarker is thus evaluated solely on internal contrast rather than external discrimination. Furthermore, given the co-linearity of H3K18la with serum CA19–9 and CEA, its incremental clinical value remains unquantified.

Several clinical variables such as metastasis (*p* = 0.0562) and histological grade (*p* = 0.0614) trend toward significance but fall below standard alpha thresholds. Given the small sample size, the study is underpowered to assess these relationships reliably. The decision to dichotomize continuous variables like tumor size (≤ 4 cm vs. > 4 cm) further reduces granularity. Such binarization obscures potential nonlinear associations, particularly in exploratory biomarker studies.

Mechanistically, the authors omit consideration of the Warburg effect ‒ the increased reliance on aerobic glycolysis in cancer cells ‒ which could independently elevate lactate levels and nonspecifically increase H3K18la. The authors also fail to account for confounders such as tissue hypoxia, which can upregulate histone modifying enzymes via HIF-1α pathways and influence lactylation indirectly^5^. Moreover, as lactate is a systemic metabolite influenced by comorbid conditions (e.g., diabetes, hepatic dysfunction), its local tissue concentration may not directly reflect tumor biology, making H3K18la a potentially nonspecific readout^6^.

Finally, there is a theoretical inconsistency in treating H3K18la both as a surrogate for systemic lactate and an independent tumor marker. If lactate is elevated in all glycolytically active tissues, then lactylation may also occur in non-malignant inflammatory or metabolic conditions, limiting specificity. Without demonstrating tissue-specific patterns, or excluding expression in non-tumor contexts, the proposal of H3K18la as a diagnostic biomarker is premature.

In summary, while the concept of histone lactylation as a molecular surrogate of glycolytic activity in pancreatic cancer is provocative, the present study is constrained by sample size limitations, statistical fragility, non-standard biomarker thresholds, and insufficient mechanistic depth. Future research should adopt prospective multi-cohort validation, integrate transcriptomic correlation with H3K18la enrichment (via ChIP-seq or CUT&RUN), and benchmark diagnostic performance against existing markers in diverse clinical scenarios.

## Declaration of generative AI and AI-assisted technologies in the writing process

### Statement

During the preparation of this work the author(s) used ChatGPT-4o in order for language, grammar, and stylistic refinement. These tools had no role in the conceptualization, data analysis, interpretation of results, or substantive content development of this manuscript. All intellectual contributions, data analysis, and scientific interpretations remain the sole work of the authors. The final content was critically reviewed and edited to ensure accuracy and originality. The authors take full responsibility for the accuracy, originality, and integrity of the work presented.

## Declaration of competing interest

The authors declare no conflicts of interest.
